# Towards Integrated Pest and Pollinator Management in Intensive Pear Cultivation: A Case Study from Belgium

**DOI:** 10.3390/insects12100901

**Published:** 2021-10-02

**Authors:** Tim Belien, Stijn Raymaekers, Maxime Eeraerts, Veerle Mommaerts, Gregor Claus, Christian Bogen, Niels Piot, Guy Smagghe, Pieter Spanoghe, Dany Bylemans

**Affiliations:** 1 Zoology Department, Research Centre for Fruit Cultivation (pcfruit npo), Fruittuinweg 1, B-3800 Sint-Truiden, Belgium; stijn.raymaekers@telenet.be (S.R.); dany.bylemans@pcfruit.be (D.B.); 2 Laboratory of Agrozoology, Department of Plants and Crops, Ghent University, Coupure Links 653, B-9000 Ghent, Belgium; maxime.eeraerts@gmail.com (M.E.); niels.piot@ugent.be (N.P.); guy.smagghe@ugent.be (G.S.); 3 Department of Horticulture, Washington State University, Northwestern Washington Research and Extension Center, 16650 State Route 536, Mount Vernon, WA 98273, USA; 4 Bayer AG, 40789 Monheim am Rhein, Germany; veerle.mommaerts@bayer.com (V.M.); christian.bogen1@bayer.com (C.B.); 5 Laboratory of Crop Protection Chemistry, Department of Plants and Crops, Ghent University, Coupure Links 653, B-9000 Ghent, Belgium; gregor.claus@ugent.be (G.C.); pieter.spanoghe@ugent.be (P.S.); 6 Department of Biosystems, KU Leuven, Decroylaan 42, B-3001 Heverlee, Belgium

**Keywords:** insect pollination, biological control, *Pyrus communis*, *Osmia* spp., mason bees, mixed hedgerow, natural enemies, ecosystem services

## Abstract

**Simple Summary:**

Over the past decades, Integrated Pest Management (IPM) strategies have been widely adopted in commercial fruit production in Europe, supporting natural pest control as an ecosystem service. At the same time, there has been a growing awareness of the importance of pollinating insects, leading to the concept of Integrated Pest and Pollinator Management (IPPM). Here we present the outcomes of a 4-year case study as a valuable illustration of an IPPM strategy in a commercial intensive pear orchard. We show how the added-value of local biodiversity measures can be visualized in front of growers, linking ecological measures to economic benefits. This scientifically-based as well as practice-oriented demonstrative case study supports the acceptance and adoption of IPPM principles in commercial intensive pear production cultivation.

**Abstract:**

Recently, the concept of Integrated Pest Management (IPM) was further extended into Integrated Pest and Pollinator Management (IPPM). Implementation of IPPM strategies entails the combination of actions for pest and pollinator management providing complementary or synergistic benefits for yield and/or quality of the harvest. The aim of this study was to examine IPPM elements (i.e., mixed hedgerow, nesting boxes for mason bees, *Osmia* spp.) and demonstrate their impact in the practical context of modern commercial fruit cultivation in a 4-year case study in an intensive ‘Conference’ pear orchard. The outcomes of visual observations during transect walks and molecular analysis of pollen collected by mason bees, showed the importance of additional floral resources for the presence of mason bees and other pollinating insects in the orchard environment. Pear quality assessments indicated that insect-mediated pollination had a significant positive impact, with a tendency for higher quality pears in the close vicinity of *Osmia* nesting boxes. However, despite the fact that pear pollen was also detected in *Osmia* spp. nest cells, the amount and frequency of pear pollen collection for their nest built-up turned out to be rather low. In the same intensive pear orchard studied for pollination effects, we simultaneously demonstrate the impact of a mixed hedgerow to enhance integrated pest control.

## 1. Introduction

The European Green Deal expresses a strong political will to design a food system that is fair, healthy, and environmentally friendly [1]. The consumption of excessive amounts of natural resources, contributions to biodiversity loss, environmental pollution and climate change and food waste have been identified as major challenges of food production while taking into account in parallel the quality of diets [1]. Consequently, sustainable agricultural production systems need to accommodate many challenges that include simultaneously support of high-quality diets, food safety, food security and the environmental footprint of its production, among others.

Fruits are an important component of healthy and sustainable diets that promote human health and wellbeing [2]. However, fruit orchards in temperate areas are often among the most intensively managed crop systems [3]. They are also an example of the variety of agronomic challenges to keep pests and diseases below economic thresholds and of the challenges of applied research efforts to identify specific biological key measures controlling pests of concern.

In Europe, fruit growers are generally familiar with the concept of Integrated Pest Management (IPM). Though historically emerging from insect pest control, IPM also refers to the management of all pest complexes including weeds and diseases [4]. IPM is a science-based decision tool that brings together preventive measures, monitoring and control with biological, physical, and chemical control agents based on warning systems [4]. The goal of IPM is to protect the health of the crop while agro-ecosystems should be the least possibly disrupted. In addition, natural pest control mechanisms are encouraged [5], which can be considered as ecosystem services. Natural pest control is generally supported by measures to protect and enhance beneficial organisms [3,4]. The pollination service of wild pollinating insects is significant for the production of fruit crops [6,7,8]. Therefore, the concept of IPM can be further extended into Integrated Pest and Pollinator Management (IPPM) [9,10,11].

### 1.1. Sustainable Production Systems Take into Account Ecosystem Services

Ecosystem services can be generally described as benefits that people obtain from ecosystems [12]. For orchards, six relevant ecosystem services have been identified, namely fruit production, climate regulation, soil nitrogen availability, water regulation, pest and disease control and pollination [13]. In this paper, a fruit orchard production system was investigated, where simultaneously several ecosystem services have been actively included as part of an integrated practice example. These focus ecosystem services include pollination and natural pest control with their effects on yields.

In pear orchards, proximity of (mixed) hedgerows positively affects beneficial arthropod species richness and abundance, indicating the potential of hedgerows as important habitats for beneficials that could reduce crop pest populations [14]. However, yields, a major and decisive aspect of applied agronomic research, were not directly considered in those investigations.

As example of successful delivery of the ecosystem service pollination, there are cases where an additional introduction of specific pollinators such as bumblebees into fruit orchards resulted in a higher mean profitability of the farm [15]. Consequently, recently target values for visitation rates of pollinators have been published to maximize crop yields, which have been developed on the basis of crop-pollinator interaction studies [6].

Generally, an investment in nesting places and supportive habitats for wild pollinators can support certain ecosystem service such as pollination. Although the majority of research in orchards concludes a positive impact [3,16], such protection and enhancement measures can also have no or even a negative effect. For instance, while non-crop habitats can increase biodiversity and enhance natural pest control they can also harbor crop pest species and therefore provide ecosystem disservices [17]. It therefore needs to be taken into account that specific IPM measures can result in both synergies and trade-offs for biodiversity. As an example for pear orchards, mechanical weed control methods such as mowing can substantially influence the number of natural enemies but also of potential prey of predators, as both can increase with a reduced mowing intensity [18]. There are also functional groups that have the potential to deliver several ecosystem services simultaneously. For example, syrphid flies could be used to increase both the ecosystem services pollination and biological pest control [19]. However, efficient biological control in fruit orchards is challenging, especially in terms of reaching sufficiently high control levels via predation and parasitism [20].

Overall, during a pear growing season, a great variety of pest insects, diseases (fungal, bacterial, and viral) and voles, mice or other vertebrates can have a devastating impact on both fruit production and quality as well as on tree vitality. To meet these challenges, fruit growers have a number of different tools available that can be used in an integrated manner to obtain a sustainable crop system. These tools range from choosing varieties that are appropriate for the regional climate and growth conditions towards optimally taking into account the various ecosystem services when planning interventions. Our study presents an example of an enhanced fruit production system with multiple benefits in terms of ecosystem services and biodiversity.

### 1.2. From IPM to IPPM in a Practical Context

Nowadays IPM control practices are widely adopted in commercial fruit cultivation in Europe. Over the past decades, IPM strategies considerably reduced the previously used calendar sprayings of agrochemical-based crop protection products, enhancing natural pest control by increasing the presence of beneficial predators and parasitoids in orchards [21]. However, IPM is not necessarily explicitly ‘pollinator friendly’ [22]. While many IPM measures taken for the purpose of pest control also have a beneficial effect on pollinators, they can have a neutral effect or even a detrimental effect as well. For instance, flower borders and/or hedgerows in field margins supplying pollen and nectar for natural enemies are often also valuable for pollinating insects and hence pollination services [10,23,24], although this is not always the case [23]. On the other hand, the expanding use of physical control measures, for example netting to exclude pest insects, can have a serious negative impact on pollinating insects [25,26]. In order to facilitate synergies between pest control practices and pollination services, the concept of IPPM was introduced and a systematic framework for this concept was recently proposed [9,11,22]. Implementation of IPPM involves the combined use of one or more intercompatible actions for pest and pollinator management, with giving priority to the actions that aim to prevent pest- and pollinator-imposed yield gaps in the long run. Consequently, the development of IPPM strategies entails the combination of actions that provide complementary or synergistic benefits for yield, and at the same time mitigating potential conflicts (e.g., ecosystem ‘disservices’) [22,27]. Designing optimal IPPM strategies will inevitably require deliberation on compatibility—a property that tends to be highly crop specific. Field trials aiming to fill knowledge gaps in this context, preferentially linked to direct practical demonstration, are a key factor in encouraging farmers and advisers to take up new practices or concepts [22]. However, crop-specific strategies managing multiple ecosystem services remain scarce.

### 1.3. Integrated Pollinator Management: Measures for Pollinators

Honey bees (*Apis mellifera* L.) are undoubtedly the best known and the most widely used managed pollinators for (fruit) crops [28]. However, flowers of pears, in particular the cultivar ‘Conference’, are not very attractive for honeybees, because they produce nectar with a low level of hexose dominant sugars [29,30]. Most cultivated pear varieties are self-incompatible, and although some cultivars can produce fruit through parthenocarpy, fruit-set and fruit quality generally benefits from cross-pollination [31,32]. The decisive role of (insect-mediated) cross-pollination for the quantity and quality of the harvest in apple is widely accepted [33,34], but has also been indicated for pear orchards by several studies [35,36,37]. Instead of honey bees, also other pollinating insects such as bumble bees (*Bombus* spp.), hover flies (syrphid flies), and solitary bees can enhance pollination services, and hence, fruit quality, fruit yield, and yield stability through time [38,39,40,41]. The presence and activity of some of these solitary bee species can also be managed to a certain extent. Especially mason bees (*Osmia* spp.) are interesting in this context, as nesting facilities can relatively easily be provided by growers [42,43]. They are known as effective pollinators of—in particular early-flowering—fruit trees [43,44], due to their capability of flying at relatively low temperature, low solar radiation, and higher wind speed and their relative short foraging distance (making them stay and actively foraging in the crop itself), and their tendency to move more between trees and tree rows compared to honey bees (improving cross-pollination from pollinizer trees planted in separate rows) [40,45,46].

Optimizing the (combined) use of individual IPPM elements and elucidating their impact on yield is a huge challenge for the organization of practical field trials. In the end, it is key to deliver outcomes that are comprehensible to the grower. Ideally, large-scale parameterized ecological studies are carried out for this purpose, in which the correlation with yield data is established. However, it is very difficult to quantify the effect of different measures embedded in different environmental conditions, let alone demonstrate them to growers. Moreover, this should be done within the economic constraints of the specific farm situation, allowing growers a certain degree of flexibility to make economically-based decisions [10]. In this article we give an example of such a grower-understandable approach by means of a practical case study in a pear orchard over four consecutive years to illustrate the potential added value of certain IPPM measures. More specifically, our research aims were to map the pollinating insects in various environments in and adjacent to the orchard such as a mixed hedgerow, and to find out whether the pollen collected by mason bees in such a biodiverse orchard environment actually contains pear pollen. Furthermore, we also aimed to determine the contribution of pollinating insects on pear quality, and the impact of (close presence) of mason bee nesting boxes herein. A last objective was to evaluate the role of a mixed hedgerow as source/refuge area of beneficial arthropods for integrated pest control.

## 2. Materials and Methods

### 2.1. Case Study Farm Site: A Modern Low-Stem Intensive Pear Orchard

**Figure 1:**
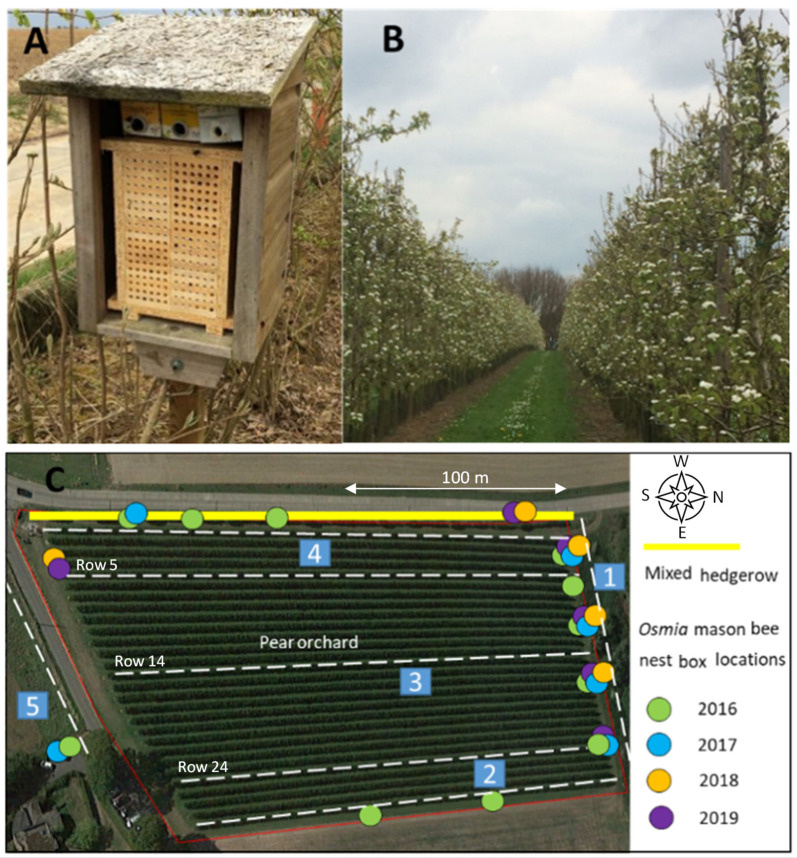
Overview of the study location (**A**) nesting box for mason bees used in the orchard; (**B**) blooming pear orchard; (**C**) schematic overview map of the orchard (ca. 200 m × 100 m, 2 ha) and the seven transect walks (dotted lines). 1: one transect walk alongside a forest area; 2: one transect walk alongside the field border; 3: three transect walks in the pear orchard (rows 5, 14, and 24); 4: one transect walk alongside a mixed hedgerow; and 5: one transect walk in a flower strip.

This case study was executed in a pear (*Pyrus communis*, cultivar Conference) orchard (ca. 200 m × 100 m, 2 ha) (Figure 1), of the Bayer ForwardFarm “Hof ten Bosch” at Huldenberg, Belgium (N 50°48′27.68″, E 4°34′20.76). The farm is characterized by sandy loam soils and an oceanic, temperate climate. When necessary, the orchard can be irrigated using pilot drip systems and frost protection sprinklers. Besides the management of pollinators and beneficials for natural pest control, the integrated crop management practices performed in the orchard include pest and disease monitoring using traps and warning systems. Weeds are controlled using both chemical and mechanical methods. Insects are controlled with mating disruption and targeted insecticide applications. Broad spectrum insecticides are only used either early in the season when beneficial arthropods are not yet present in the orchard or as correction spray in case of high pest pressure resulting in economic losses. For fungal disease control, fungicides are applied. Fertilization takes place via soil and leaves. Timing of interventions and further details are listed in Supplementary Table S1.

The study orchard is situated in an intensively managed agricultural landscape. Semi-natural habitat elements were present in the form of an adjacent forest patch (ca. 50 × 100 m, 0.5 ha) and a mixed hedgerow at the west side (ca. 200 m × 3 m, 0.06 ha). The forest area comprised several wild plant species, among others: *Epilobium hirsutum* (great hairy willowherb), *Heracleum sphondylium* (common hogweed) and *Rubus fruticosus* (blackberry). The mixed hedgerow was composed of 15 different woody plant species (for the exact composition, see Supplementary Table S2). In addition, a flower strip was sown in the grassland next to the orchard in 2016 (Figure 1). The flower mix contained among others *Phacelia tanacetifolia* (lacy phacelia), *Papaver* spp. (poppies), and *Vicia* spp. (vetches). Other entomophilous crops were cultivated in the immediate vicinity (*Brassica napus* subsp. *napus* (oilseed rape) in 2017, and *Trifolium* spp. (clover)). Within a 250 m radius, 66% of the land cover was classified as intensive agriculture (winter wheat (*Triticum* spp.), winter oilseed rape, potato (*Solanum tuberosum*), sugar beet (*Beta vulgaris*), maize (*Zea mays* subsp. *mays*)), 12% as permanent pastures and 2% as forage crops (temporary grassland: mixture of perennial ryegrass (*Lolium perenne)* and white clover (*Trifolium repens*)). The remainder of 22% consisted of semi-natural elements, gardens, paved surfaces, roads, and infrastructure [47]. Within the orchard, several floral resources were available: primarily pear blossoms and secondarily weed flowers (among others: *Bellis perennis* (common daisy), *Cirsium arvense* (field thistle), *Lamium purpureum* (purple dead-nettle), *Taraxacum* agg. (common dandelion)).

During the study period (2016–2019) several nesting boxes for mason bees were provided at different locations at the borders of the orchard (Figure 1). Nesting boxes were built with 12 layers, each with 12 U-shaped tunnels (8 mm), made from softwood timber (*Picea abies*). The layers were retained in an easy-to-open moisture resistant medium density fiberboard casing, allowing multiple samples to be taken throughout the season.

### 2.2. Plant-Pollinator Observations via Transect Walks

Data of flower-visiting insects was collected by transect walks after pear tree blossoming, in three consecutive years (2016–2018). In short, observations were performed when weather condition would allow optimal pollinator activity: air temperature of at least 13 °C, no rain, cloud cover <50%, and a low wind speed (maximum 3 m/s). Each transect (see Figure 1C) was executed with a slow walking speed (ca. 1.2 km/h) during 10 min. On each observation day, transect walks were done in the morning (10:00–12:00 h) and afternoon (14:00–16:00 h).

During the transect walks, flower-visiting bees and hover flies were caught with an insect net and individually stored in polypropylene centrifuge tubes (Greiner Bio-One BVBA, Vilvoorde, Belgium). This prevented counting the same individual twice. The plant species on which the insect was observed was recorded. After the transect walk, identification of insect to species level was immediately performed if possible, and the insect was released. When identification in situ was not straightforward, samples were taken for identification using species reference guides. Insect species were afterwards attributed to broad functional pollinator groups. These groups were: honey bees (*Apis mellifera*), bumble bees (*Bombus* spp.), solitary bees (all other species of Apoidea, Hymenoptera), and syrphid flies (species of Syrphidae, Diptera).

In 2016 and 2017, insect observations were also made during the pear blooming period. In 2017, weather conditions during flowering at the moments of pollinator observations were very poor (windy, rainy, cold), so no relevant and reliable data could be obtained.

### 2.3. Microscopic Analysis of Solitary Bee-Collected Pollen

In May of 2017 and 2018 (after the pear blossoming and *Osmia* nest construction period), pollen was collected from 10 random brood cells of 4 random *Osmia* nesting box modules (hence, in total each year 40 samples). After storage of the samples in 70% ethanol in Eppendorf tubes (VWR International BVBA, Heverlee, Belgium), each sample was vortexed, and 5 µL was deposited on a pre-cleaned glass microscope slide (76 × 26 × 1 mm). A drop of fixative (a mixture of glycerol and the dye eosin blue, heated to 55 °C) was added and the droplet was covered with a standard cover slip (24 × 32 mm). For each sample, 100 randomly chosen pollen grains were identified up to the plant family level using a microscope (ZEISS Axiovert 25C, Carl Zeiss NV, Zaventem, Belgium).

### 2.4. Molecular Analysis of Solitary Bee-Collected Pollen with Pear-Specific Primers

To detect the presence of *Pyrus communis* pollen in the brood cells of the *Osmia* spp. a pear-specific primer set was designed (forward: 5′-CYCGAKAACCYRTTCCRAYKTCG-3′; reverse: 5′-TATYCRTTRCYRAGWGTHRTTTTGAC-3′; amplifying a ~220 bp fragment) [ordered at Integrated DNA Technologies, Coralville, Iowa, United States]. Primers were designed using BioEdit (Version 7.0.5.3) [48] based upon a dataset (NCBI accession: 380855378) published by Lo et al. [49] containing the partial internal transcribed spacer (ITS) 1, complete 5.8S ribosomal RNA gene and partial internal transcribed spacer 2 sequences of multiple common Rosaceae.

The specificity of the designed primer set was tested on leaf-extracts of apple (*Malus domestica*), cherry (*Prunus* spp.), and pear (*Pyrus communis*) as well as on five pollen mixtures (originating from *Osmia* spp. brood cells with known plant genus composition [50] (see supplementary Table S4)). The primers showed no cross reactivity and could detect *Pyrus communis* in the mixed pollen sample. The PCR analysis (SensoQuest, Göttingen, Germany) of the extracted samples was as follows: each 25 µL PCR reaction contained 2.5 µL 10X PCR buffer, 1 µL 50 mM MgCl_2_, 0.75 µL dNTP (10 mM), 0.25 µL Taq Polymerase (5 U/µL) (Thermo Fisher Scientific, Waltham, MA, USA), 5.5 µL nuclease free water, 5 µL Forward and 5 µL reverse primer and 5 µL sample. PCR conditions were as follows: 4 min 95 °C followed by 36 cycles of 40 s 95 °C, 40 s 56 °C and 40 s 72 °C and ended with 5 min elongation at 72 °C. PCR results were visualized on a 1.5% agarose gel and stained with ethidium bromide. All PCRs were run with the appropriate controls to exclude false negatives or false positives. Furthermore, positive samples were sent for Sanger sequencing (LGC Genomics, Middlesex, United Kingdom) to confirm the identity of the detected fragment. A total of 10 pooled pollen samples collected across 3 years was analyzed for the presence of pear (2018 (*n* = 1), 2019 (*n* = 5) and 2020 (*n* = 4)). Brood cell pollen from separate nesting cavities and modules was collected from the trap-nest and mixed.

Both leaf-extracts and pollen were extracted in a similar manner. Ca. 1 cm^2^ of fresh leaves of *Malus domestica*, *Prunus* spp. and *Pyrus communis* were ground in liquid nitrogen with a mortar and pestle. Ground material was transferred to a sterile 2 mL tube. Pollen samples were homogenized with mortar and pestle and approximately 0.07 g of homogenized sample was transferred to a 2 mL tube. Further extraction proceeded as described earlier [50], using the Invisorb Spin Tissue Mini Kit (Stratec Biomedical, Birkenfeld Germany). Extracted DNA was stored at −20 °C until further use. To test the DNA extraction success, a PCR with broad range primers detecting the ITS2 region of plants was used, as described earlier [51]. All extractions were successful.

### 2.5. Contribution of Insect Flower Visitation and Impact of Mason Bees Nesting Boxes on the Fruit Quality

During four subsequent years (2016–2019), fruit set and fruit quality were measured. Each year, 15 trees spread over 3 different sample sections (over rows 5, 14, and 24) (Figure 1) were selected and marked according to 3 different distance classes from the present nesting boxes for mason bees: 0–10 m, 10–50 m, and 50–100 m. Before flowering, 5 branches of the marked trees were assigned to two pollination treatments: one with sleeves (mesh size 1 mm × 1 mm) (flowers excluded from insect visitation, hereafter denoted as “bagged” treatment), and another part of branches was marked but not covered (flowers open for insect visitation, hereafter denoted as “open” treatment). After flowering of the pear trees, the sleeves were removed. At harvest, all pears from the open and bagged treatment were counted and harvested. For every pear, the weight, diameter, and length were recorded. From this data, a ‘quality-index’ was calculated according to the formula: quality-index = diameter/length × weight (see overview of the number of pears for which the quality-index was determined in Table 1). Pears with a quality-index below 75, as a consequence of a bottle-like shape (=low diameter/length ratio) and/or a low weight, are considered of inferior quality. The resulting data were analyzed with linear regression modelling in RStudio (version R 3.6.2). Appropriate use of models and the required assumptions for linear regression were checked through diagnostic residual plots and the Shapiro-Wilk test for normality. The outcome variable ‘quality-index’ was square-root transformed to assure normality. For the first research objective, in which the impact of flower visitation by insects in general on the fruit quality of the pears was analyzed, the square-root transformed pear quality-index was modelled with the pollination treatment (open vs. bagged treatment) and year as interaction terms (model = lm(sqrt(quality-index) ~ pollination treatment*year). For the second research objective, in which the impact of the presence of *Osmia* nesting boxes on the fruit quality of the pears originating from the open treatment was analyzed, the square-root transformed pear quality-index was modelled with the distance to the *Osmia* nesting boxes and year as interaction terms (model = lm(sqrt(quality-index) ~ distance*year). Post hoc Tukey multiple and pairwise comparisons were performed using the R *emmeans* package (version 1.4.5) at minimum significance level α = 0.05.

### 2.6. Mixed Hedgerows as Source/Refuge Area of Beneficial Arthropods

During 3 subsequent years (2016–2018) a beating sampling protocol was executed at 4–6 different sampling days during the season: 1–2 samplings during April (early spring, before flowering of pear trees), 1–2 sampling during May–first half of June (spring, after flowering period) and 2 samplings between second half June and September (summer-harvest period). In the morning of each sampling date 15 different trees of the mixed hedgerow as well as 15 trees randomly selected in the pear orchard (Figure 1) were sampled by the limb beating method. For each sample 3 branches were beaten 3 times, and all falling arthropods were collected for subsequent identification to family/species level. Based on their known role, the collected arthropods were divided into three classes: pest, beneficial (predator and parasitoids of pest species), and indifferent species (all arthropods with no known pest or beneficial characteristics). All count data were analyzed with a Generalized Linear Mixed Model using the *glmmTMB* package in R. A zero-inflated GLMM with Poisson distributed errors was used to model the beneficial counts with ‘Habitat’ (hedgerow or orchard) and ‘Timing in season’ (early spring (before flowering), spring (after flowering period) and summer) as fixed factors with their corresponding interaction, and ‘Tree’ (plant species in mixed hedgerow/orchard) and section of the sampled tree in the north-south orientation (parallel with the hedgerow) as random intercepts. Appropriateness of the model was assessed through diagnostic residual plots and through evaluation of dispersion using the *DHARMa* package. Post hoc Tukey multiple and pairwise comparisons were performed as described above.

## 3. Results and Discussion

### 3.1. Plant-Pollinator Observations

**Figure 2:**
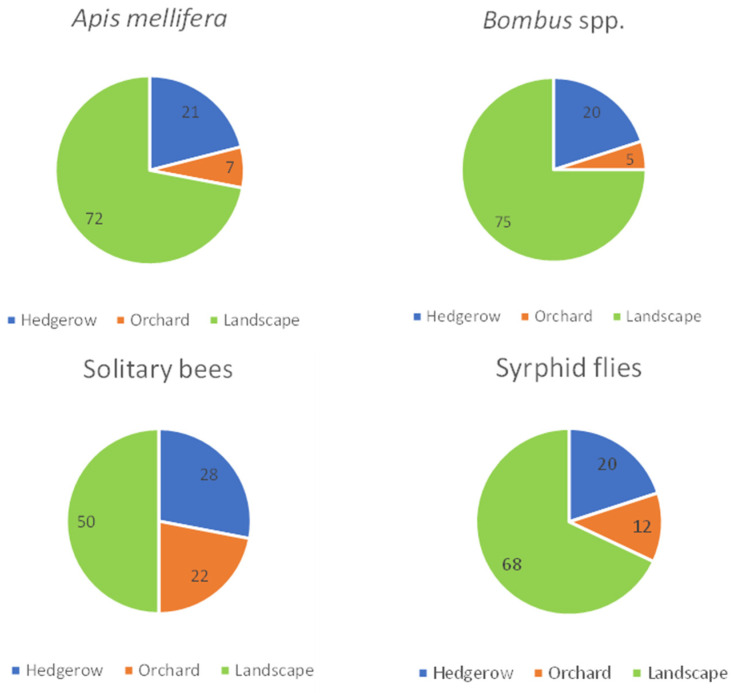
Overview presence of functional group in the different environments (hedgerow, orchard, landscape). Data are presented as average % of three consecutive monitoring years (2016, 2017, and 2018).

During the 2016 pear flowering period about one third of all pear flower-visiting insects were observed to be *Osmia cornuta* (see Supplementary Table S3A). After pear flowering, a variety of flower-visiting insects were observed during the transect walks in 2016, 2017, and 2018 (a detailed list of the identified species is provided in Supplementary Table S3B). Although *Osmia bicornis* as well as *Osmia cornuta* mason bees were found to occupy the nesting box modules, only *O. cornuta* was observed during the transect walks, both on pear trees and other plants. The different functional groups monitored were present in the three environments (see Figure 1: orchard: transects 2 and 3, mixed hedgerow: transect 4 and landscape: transects 1 and 5). The floral resources in the habitat elements in the adjacent landscape showed the highest attractiveness followed by the hedgerow for all functional groups. The supportive role of the orchard was most pronounced for the solitary bees followed by syrphid flies, *Apis mellifera* and *Bombus* spp. (Figure 2). A detailed list of the plants visited for each functional group over the different years is listed in Supplementary Table S4.

Plants such as *Taraxacum* agg. and *Trifolium repens* were regularly visited in the orchard and this is in agreement with Eeraerts et al. 2021 [52] who determined that these are characteristic plants species for the herb layer of alleyways in fruit tree orchards. These weeds, like many non-intentionally sown plants, provide an important food source for bees and other pollinating insects [53]. Due to the use of pesticides in orchards, these weeds could potentially be in contact with pesticide residues and thus expose bees to pesticides. Nonetheless, determination of residues, ecotoxicological tests on representative species and calculation of different exposure scenarios are part of testing and risk assessments in the pesticide (referred to as plant protection product) registration context within the European Union (e.g., Reg. 283/2013 and 284/2013 concerning the data requirements, Guidance Document on Terrestrial Ecotoxicology, SANCO/10329/2002, rev. 2 final, 17.10.2002 concerning risk assessments). Corresponding reports that relate to the active substances and representative formulations with more detailed information on their safety and effects profile, including risk mitigation, can be accessed via the EFSA homepage [54]. However, the general assessment of the environmental effects of combined management practices beyond solely chemical products in applied agricultural contexts, considering the yield quantity and quality on those insect groups have not been studied much. Given the higher diversity of attractive plants in the hedgerow, the flower strips and the natural habitat in the area, it is obvious that we observed more pollinators in these environments compared to the orchard environment. Landscape features such as hedgerows, forest edges, and field margins often provide a higher diversity and abundance of floral resources for bees and other pollinating insects throughout the year compared to agricultural land [52,55]. By collecting plant-pollinator data in these elements, we expand our knowledge of which plant species potentially support a wide diversity of beneficial insects such as pollinating insects. Plants such as *Cornus sanguinea*, *Frangula alnus,* and *Cytisus scoparius* are clearly useful food sources and provide a labor-extensive opportunity to further support key pollinating insects for crop production after flowering of the fruit crops.

### 3.2. Analysis of Osmia-Collected Pollen

• Microscopic analysis

In 2017, the percentage of Rosaceae pollen varied from 0.9 ± 1.4% (SD) to 19.3 ± 22.8%, and in 2018 from 19.6 ± 25.6% to 50.1 ± 27.8% (for the nesting box module with the lowest and highest presence of Rosaceae pollen, respectively). Since the mason bee nesting boxes modules were cleaned and installed just before the pear flowering period, and the pollen was sampled from the nesting boxes just after the pear blossoming and nest construction period, the investigated pollen was collected by the mason bees during the pear flowering period. However, pollen of Rosaceae (including pear trees *Pyrus communis*, but also other plants such as cherry trees *Prunus avium,* which were present in the adjacent garden) cannot be distinguished from each other using a light microscope, so for further detailed identification molecular analysis was needed (see below). The difference in the proportion of Rosaceae pollen between 2017 and 2018 is most likely caused by the much better conditions (sunny, windless weather) for pollination during the full bloom phenological stage of pear in 2018 compared to 2017, favoring the flying and flower visiting activity by the mason bees during pear blossoming in 2018.

• Molecular analysis

In this study we designed pear-specific primers which could readily detect the presence of pear pollen in mixed pollen samples and showed no cross-reactivity with other fruit crops from the same family (i.e., apple and cherry) and pollen from other frequently visited plant species by *Osmia* spp. (see Supplementary Table S4). This developed primer set could be useful in future studies to detect the presence of pear in mixed pollen samples.

Within the analyzed pollen samples (*n* = 10) collected over a three-year period at the Huldenberg experimental farm we found that 10% contained pear pollen, i.e., only in the sample collected in 2018. This result is in line with the result of a previous study [50]. In that study, pollen collected by *Osmia* spp. was analyzed from trap nests placed in the vicinity of pear orchards, similar to the current study. The authors found that only ca. 5% of the analyzed pollen samples (*n* = 174) contained *Pyrus communis*. The detection of pear pollen in our study is a confirmation of the field observations (done by transect walks) that show that *Osmia* spp. visit the flowers of pear trees, however the amount and frequency of pear pollen collection for their nest built-up appears to be relatively low. Other managed pollinators such as honey bees (*Apis melifera*) or bumble bees (*Bombus* spp.) could therefore be a valuable additional IPPM element to further improve the pollination, yield, and pear quality. After all, honey bees were found to be the most frequent visitors of Conference pear flowers in our study (see Table S3A) as well as in another study from the UK [37]. Bumble bees were earlier observed to fly very intensively from flower to flower, visiting half-open and even closed flowers as well [56]. However, in the latter study it was also noticed that bumble bees were too active, damaging the flowers and negatively impacting fruit set, at least in caged pear trees. Moreover, although managed bees can be a crucial factor in orchards in ensuring sufficient pollination, one should also bear in mind that there is a risk of possible negative effects of these managed bees on wild bee abundance and diversity, caused by e.g., competition or shared antagonists such as pathogenic organisms [57].

### 3.3. Contribution of Insect Flower Visitation to the Fruit Quality

**Figure 3:**
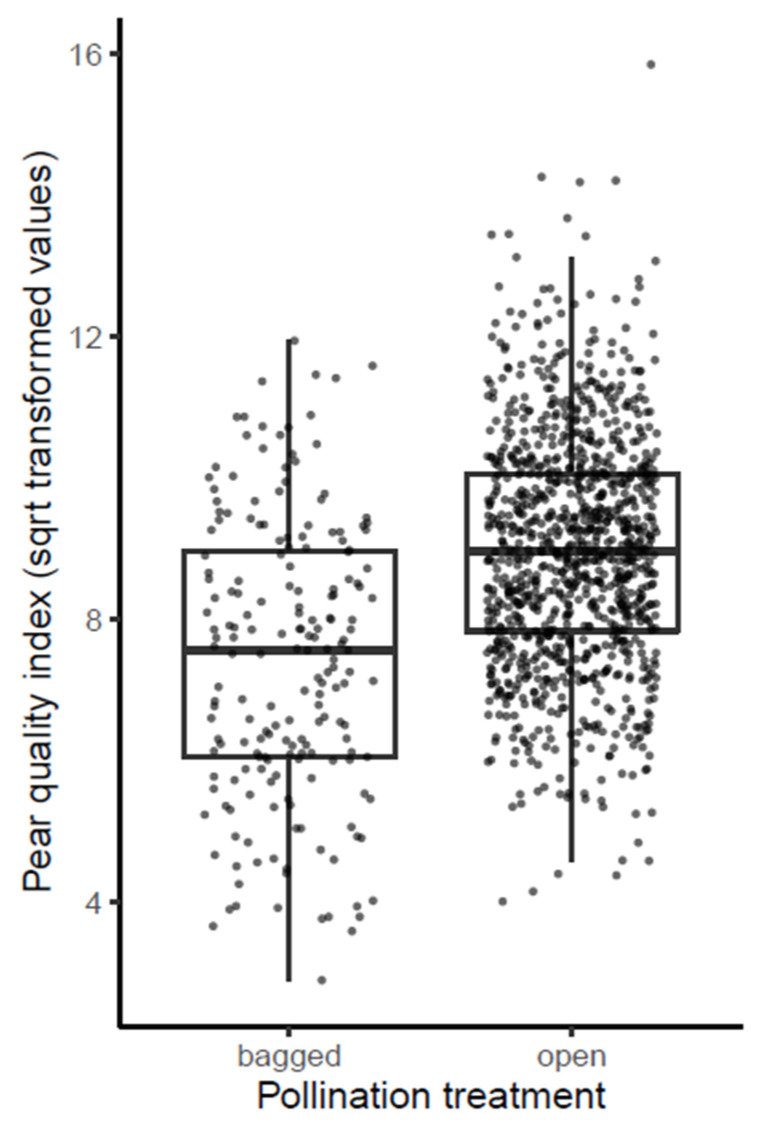
Pear fruit quality-index (=diameter/length × weight) for the different pollination treatments (bagged = flowers excluded from insect visitation, open = flowers open for insect visitation). Results are from linear regression models with square-root transformed quality-index values with pollination treatment and year as fixed factors. Individual data points are shown as little black dots and summarized as box plots.

With respect to the third research objective, we analyzed the impact of flower visitation by insects in general on the fruit quality of the pears. Because the data of 2016 did not include the bagged treatment, these were excluded from this analysis. Pears originating from flowers from the open treatment were in general of higher quality than pears from the bagged treatment (overall mean quality-index of 83.1 ± 30.1 vs. 58.9 ± 29.6, respectively, Table 2; Figure 3). In the linear regression modelling analysis, the independent variables pollination treatment, year as well as their interaction were identified as significant predictors of the quality-index (Table 2).

The significant impact of the factor year reflects the overall differences in pear quality between the different harvest years concerned, due to differences in weather conditions, pest pressure, and other year-dependent orchard management treatments between the different years. The consequent significant effect of the pollination treatment factor during the subsequent years (*p* = 0.039, <0.001 and 0.017 in pairwise comparisons between ‘open’ and ‘bagged’ for 2017, 2018, and 2019, respectively) demonstrates that pollination by insects had a consistent positive impact on the quality of the pears. An analogous analysis in which 17 outliers were removed from the dataset delivered similar outcomes (see Supplementary Table S5). These results are in accordance with previous studies about the contribution of insect pollination to pear fruit set and quality [31,37]. Despite the fact that this pear variety Conference can form fruits by parthenocarpy and thus without pollination, there is certainly an advantage of insect pollination in terms of pear quality. Parthenocarpy induction by spraying plant hormones, mainly gibberellins, is a common practice in a number of Belgian Conference. It increases the fruit set using phytohormone treatments but does not result in larger fruit sizes and weights [31]. In the orchard and period of this study gibberellin treatments were only applied in 2016, leading to very high numbers of parthenocarpic fruits. However, this year was excluded from the above analysis.

Depending on the exact dimensions of height and weight, the difference between a mean pear quality-index of 83.1 (with insect-mediated pollination by flower-visiting insects) and 58.9 (without insect-mediated pollination by flower-visiting insects) corresponds to differences of about 10–20% in diameter size classes of pears. Based on recent figures of medium prices per size class (see Supplementary Table S6) this corresponds to a price difference of about 0.1 EUR/kg, which is considerable in economic terms. For a pear orchard with a mean production of 40,000 kg/ha this means a yield difference of EUR 4000 per ha.

### 3.4. Impact of Osmia Nesting Boxes on the Fruit Quality

**Figure 4:**
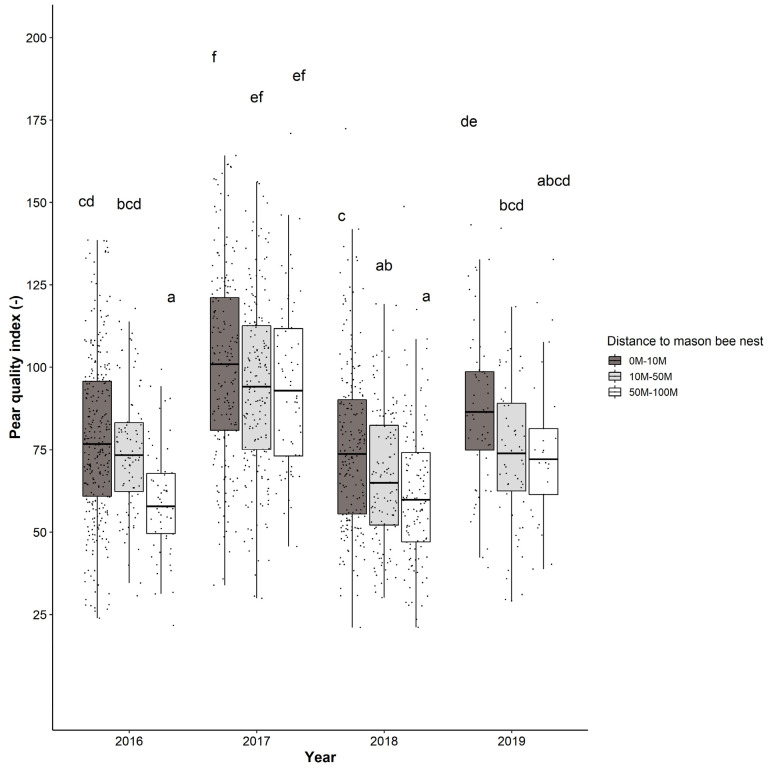
Pear fruit quality-index (=diameter/length × weight) for the different distances to *Osmia* nesting boxes for 2016–2019. Results are from linear regression models with square-root transformed values with distances to *Osmia* nesting boxes and year as fixed factors. Individual data points are shown as little black dots and summarized as box plots. Statistical differences are indicated with different letters.

In a fourth research objective we analyzed the impact of the presence of *Osmia* nesting boxes (and their distance to the flowering trees) on the fruit quality of the pears. For this analysis, the data of pears harvested from the bagged treatment were excluded, as these covered flowers could not be properly visited by mason bees or other pollinating insects. In the linear regression modelling analysis the variables’ distance to mason bee nesting boxes’ and year were identified as meaningful predictors of the quality-index (each with significance *p* < 0.001), as well as their interaction effect (*p* = 0.016; Table 3). In 2016, the quality-index of pears nearby *Osmia* nesting boxes (0–10 m and 10–50 m) was significantly higher than pears harvested from pear trees further distanced from *Osmia* nesting boxes (50–100 m) (Table 3 and Figure 4). In 2018, the pears in the closest distance region (0–10 m) were of significant higher quality (*p* = 0.038 and < 0.001 for the comparisons with 10–50 m and 50–100 m distance, respectively). Earlier studies already demonstrated the pollinating capacity of *Osmia* species on pear [58,59,60]. The impact of *Osmia* nesting boxes within a radius of 0–50 m is in line with the typical foraging range of mason bees of 50–200 m when floral resources are abundant [61]. For the other two years (2017 and 2019) no significant differences were found in the pairwise comparisons between the different distance classes. Note that in 2017 also no pear pollen was detected in the collected pollen in the nest boxes. Furthermore, in these years the mason bees’ pollination effect was presumably also averaged out by other factors. For instance, overnight frost events during the pear flowering periods (which were present in 2019, and very severe in 2017), undoubtedly had an overall substantial impact on fruit set and fruit development [62]. An analogous analysis in which 16 outliers were removed from the dataset delivered similar outcomes (see Supplementary Table S7). The overall effect of the distance to *Osmia* nesting boxes is also displayed by plots of the quality-index of the pears in relation to the different distance classes (see Figure 4). From the presentation of the results in Figure 4 it is also clear that the specific year (with many underlying factors such as weather conditions and spraying schedule) had a (significant) impact on the quality of the pears. Furthermore, limited data (not shown) on the number of healthy seeds indicated that only a small minority of the pears contained healthy seeds. Therefore, it is difficult to substantiate from our data that the positive relationship observed in 2016 and 2018 between pear quality and distance to *Osmia* nesting boxes can unambiguously be linked to the pollination activity of *Osmia* bees. If pollination activity was not the decisive factor in this, other (in)direct effects may also have played a role. For example, the nest boxes may also have been exploited by natural enemies, which in turn have had an effect on pests (e.g., aphids, scales, psyllids) and in this way could have indirectly benefited the fruit size and quality.

Over the 4-year study period the mean pear quality-index of pears harvested in the closest distance range to the *Osmia* nesting boxes (0–10 m) was 88.3, whereas for the longest distance range (50–100 m) a mean quality-index of only 73.0 was reached. These differences in quality are more or less similar to the previous analysis, and hence also correspond to an economic revenue of EUR 4000 per ha by a dense network of *Osmia* nesting boxes. In a recent study in commercial tart cherry orchards, a more equally distributed network of closer-distanced nesting boxes was also found to be in favor for propagation of mason bees (*O. lignaria*) compared to a less dense distribution of nesting boxes [63]. Mason bees, in particular *O. cornuta*, are put forward as pollinators in pears because they are more active foragers than, e.g., honey bees in the often colder temperatures during pear flowering [59]. In addition, given they have a smaller foraging radius compared to honey bees, they also stay closer to their nest once they have started a nest and are more likely to forage on the crop [64].

### 3.5. Mixed Hedgerow as Source/refuge Area of Beneficial Arthropods

**Figure 5:**
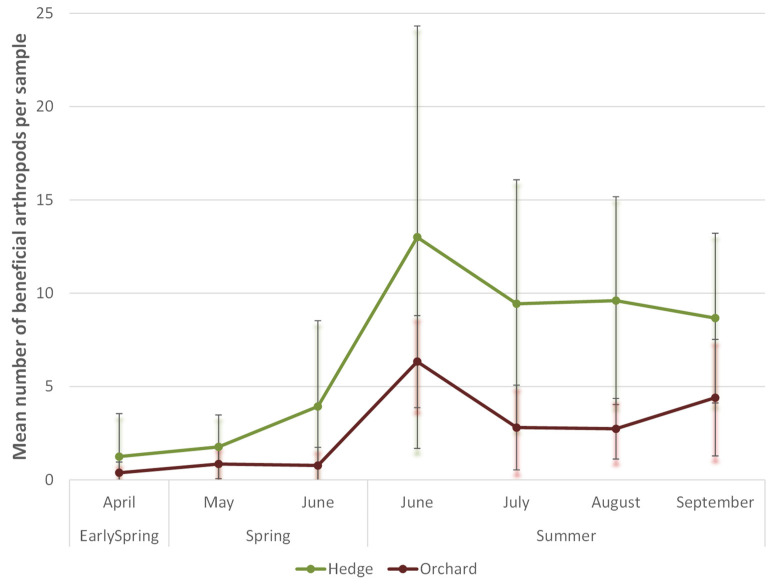
Overview of evolution of the abundance of beneficial arthropods in the mixed hedgerow and pear trees throughout the season based on mean counted numbers in collected samples in three subsequent years (2016–2018). Early spring = before flowering of the pear trees. Means and standard errors for all samples of the different years are shown in the graph.

During three subsequent years (2016, 2017, and 2018), a total of 4148 arthropods were sampled. Almost half of them (2046 specimens) were classified as indifferent, while 31% (1289 specimens) were identified as beneficial and 19% (813) as pest organisms (see Supplementary Table S8 for an overview of the identified species/families). We observed to a large extent the same species in the orchard and the hedgerow, in particularly regarding the beneficial arthropods. Therefore, the analysis was focused on this part of the dataset. Overall, clearly more beneficial arthropods were collected from the different plant species in the mixed hedgerow than from the pear trees in the orchard (overall mean of 4.8 ± 5.6 vs. 2.1 ± 2.5 per sample, respectively). Accordingly, the specific environment (hedgerow or orchard) was identified as a significant predicting factor for the presence of beneficial arthropods (*p* < 0.001). The number of beneficial arthropods strongly increased during spring in the hedgerow as well as in the pear trees, reaching overall maximum sampled numbers at the end of spring/beginning of summer (second half June) (Figure 5). Early in the season (before flowering of the pear trees) the relative difference between the number of beneficial arthropods in the hedgerow and orchard is the largest, with only a very scarce presence of beneficial arthropods in the pear trees and about 3.5 times higher numbers sampled in the hedgerow. The increase of beneficial arthropods in the hedgerow in early spring is clearly preceding the steep beneficial population built-up in the pear orchard during spring (Figure 5), with in general the lowest difference between both habitats near harvest at the end of summer (less than double of the numbers of beneficial arthropods sampled in the hedgerow vs. the pear trees). This time-dependence in the dynamics of beneficial arthropod populations between the hedgerow and the pear trees is reflected by the interaction effect between both predictors in the GLMM analysis. In early spring (before flowering) the difference in beneficial arthropod numbers between hedgerow and orchard trees was extremely significant (GLMM, post hoc pairwise test: *p* < 0.001), while later on, lower significance levels (GLMM, post hoc pairwise tests: *p* = 0.002 and *p* = 0.026 during spring and summer, respectively) were obtained. Since the microclimate in the hedge is very similar to the microclimate in the orchard, the observed differences in dynamics can in all likelihood be explained by an effectively higher overwintering rate in the hedgerow or earlier attraction of beneficial insects from the wider environment, rather than a supposedly faster phenological development of insects in the hedge compared to the orchard.

These findings demonstrate the importance of the mixed hedgerow as a habitat for beneficial arthropods who can provide pest control. After attraction and/or built-up of beneficial arthropods in the mixed hedgerow, the pear trees are colonized by beneficial populations during the post-flowering period. The colonization pattern of beneficial populations during the post-flowering period supports the ‘selection of time’ principle in modern IPM pear growing, in which correction sprays with broader range crop protection products (e.g., pyrethroids) are still possible in the pre-flowering period but should be avoided in the post-flowering period [65]. As for the case study on the effect of measures for pollinating insects, it should be noted that despite the fact that this is a multi-year study (providing us a rough picture of seasonal (weather) effects), we cannot draw any conclusions about potential landscape or environmental influences, as this case study was focused at only one location. However, it is striking that at this farm there are hardly never noteworthy problems with the pear sucker *Cacopsylla pyri*, which is by far the most devastating pest in many other pear production sites in Belgium [66]. Most likely, this can be explained by the fact that this orchard is located within a more diverse landscape with almost no other pear orchards and a lot of heterogeneous vegetation types, and other land use types, similarly as described earlier for vineyards [67]. This in contrast to major fruit production regions in Belgium with large contiguous areas (10 ha and more) of pear orchards (essentially monoculture variety ‘Conference’) in which pear psyllids in general are very poorly suppressed by their natural enemies.

The ecosystem service of pest control is generally well known by modern fruit growers [21,68]. Beneficial arthropods play a major role in the sustainable control of various pests [3,69]. Predatory arthropod communities and influences of local and landscape factors are shown to be strongly shaped by orchard management practices [70,71]. The role of local biodiversity elements such as hedgerows and flower borders has been highlighted in several studies [72,73]. Less studies are devoted to the translation of the presence of natural enemies of fruit pests into the final economic value for the fruit grower [68,74]. In the same intensive pear orchard being subject of the case study on measures for pollinating insects, we here simultaneously demonstrate the impact of a mixed hedgerow as a specific biodiversity element to enhance integrated pest control, as an example of an IPPM strategy in modern commercial pear production.

## 4. Concluding Remarks

This case study represents a valuable illustration of an IPPM strategy in modern commercial fruit growing in a low-stem intensive pear orchard. We show how the added-value of local biodiversity measures can be visualized in front of growers (linking ecological measures to economic benefits) through practice-based demonstrative research to encourage the implementation of these measures in their orchard management. Although this is a multi-year study, which has given us a rough picture of seasonal (climatological) influences, we cannot draw conclusions about other (abiotic) factors such as the influence of the nearby and wider environmental/habitat characteristics, as this case study focused on only one location. Yet, landscape and environmental factors must not be overlooked, as various recent scientific studies point to the critical role of habitats surrounding fruit orchards to sustain healthy pollinator communities, while the effect of local management generally is less consistent [75,76,77,78].

## Figures and Tables

**Table 1:** Overview of the number of pears for which the quality-index (=diameter/length × weight) was calculated over the years. Bagged = pears originating from flowers excluded from insect visitation. Open = pears originating from flowers open for insect visitation.

	Treatment				
Year	Bagged	Open Distance to *Osmia* Nesting Boxes			Total
		0–10 m	10–50 m	50–100 m	
2016	0	298	90	60	448
2017	60	189	190	62	501
2018	108	208	122	122	560
2019	24	60	61	30	175
Total	192	755	463	274	1684

**Table 2:** Results of linear regression models assessing the effect of pollination treatment (bagged vs. open flowers) (bagged = flowers excluded from insect visitation, open = flowers open for insect visitation) and year on pear fruit quality-index (=diameter/length × weight). Model statistics degrees of freedom (df), *F*-values, *t*-values, and (Tukey-adjusted) *p*-values are given.

**Factor**	**df**	***F***	***p***
Pollination treatment	1	132.7	<0.001
Year	2	190.1	<0.001
Pollination treatment:Year	2	12.6	<0.001
Comparisons *	***t***	***p***	
2017	−2.9	0.039	
2018	−11.8	<0.001	
2019	−3.2	0.017	

* Pairwise a posteriori comparison tests among the pollination treatments (bagged vs. open for each year).

**Table 3:** Results of linear regression models assessing the effect of the distance to *Osmia* nesting boxes and year on pear fruit quality-index (=diameter/length × weight). Model statistics degrees of freedom (df), *F*-values, *t*-values and *p*-values are given.

**Factor**	**df**	***F***	***p***
Distance	2	32.5	<0.001
Year	3	100.3	<0.001
Distance:Year	6	2.6	0.016
Comparisons *		***t***	***p***
2016	0–10 m—10–50 m	0.9	0.99
	0–10 m—50–100 m	−5.6	<0.001
	10–50 m—50–100 m	−4.1	<0.01
2017	0–10 m—10–50 m	2.3	0.50
	0–10 m—50–100 m	1.5	0.95
	10–50 m—50–100 m	0.2	1.00
2018	0–10 m—10–50 m	3.4	0.038
	0–10 m—50–100 m	5.3	<0.001
	10–50 m—50–100 m	1.7	0.86
2019	0–10 m—10–50 m	2.9	0.14
	0–10 m—50–100 m	2.1	0.61
	10–50 m—50–100 m	−0.3	1.00

* Pairwise a posteriori comparison tests for each year between the different distances to the *Osmia* nesting boxes.

## Data Availability

The data presented in this study are available in the supplementary material or on request from the corresponding author.

## Supplementary Materials

The following are available online at https://www.mdpi.com/article/10.3390/insects12100901/s1: Table S1. Products used and timing of interventions in the study pear orchard over the different years: A) 2016 -B) 2017 -C) 2018. Table S2. Composition of the mixed hedgerow at the border of the study pear orchard. Table S3. Overview of insects observed visiting pear flowers during pear flowering in 2016 (A), and overview of the flower-visiting insects monitored during the transect walks after flowering in 2016-2018 (B). Table S4. Overview of the plants visited for each functional group over the different years: A) 2016 -B) 2017 -C) 2018. Table S5. Results of linear regression models assessing the effect of pollination treatment (bagged vs open flowers) and year on pear fruit quality without outliers (*n* = 17). Model statistics degrees of freedom (df), *F*-values and *p*-values are given. Table S6. Medium price per size class for 'Conference' pears for the 2015-2016 season for quality class A3 (Belgische Fruitveiling BFV, 2016, personal communication). Table S7. Results of linear regression models assessing the effect of the distance to *Osmia* nesting boxes and year on pear fruit quality without outliers (n = 16). Model statistics degrees of freedom (df), *F*-values and *p*-values are given. Table S8. Overview of species/families (or a higher taxonomic level*) that were identified in the limb beating samples in the pear trees and in the mixed hedgerow, and their considered role (pest, beneficial or indifferent).
