# Supplementary material for: Towards Integrated Pest and Pollinator Management in Intensive Pear Cultivation: A Case Study from Belgium
Source: Insects. 2021 Oct 2;12(10):901. doi: 10.3390/insects12100901 (PMC8539969; doi:10.3390/insects12100901)
Supplement: Supplementary file 1 [file insects-12-00901-s001.zip › insects-1301042-SI.pdf]

insects MDPI

Supplementary Materials

Table S1. Products used and timing of interventions in the study pear orchard over the different years: (A) 2016 –(B) 2017 –(C) 2018.

Table S2. Composition of the mixed hedgerow at the border of the study pear orchard.

Table S3. Overview of insects observed visiting pear flowers during pear flowering in 2016 (A), and overview of the flower-visiting insects monitored during the transect walks after flowering in 2016–2018 (B).

Table S4. Overview of the plants visited for each functional group over the different years: (A) 2016 –(B) 2017 –(C) 2018.

Table S5. Results of linear regression models assessing the effect of pollination treatment (bagged vs open flowers) and year on pear fruit quality without outliers (n = 17). Model statistics degrees of freedom (df), F-values and p-values are given.

Table S6. Medium price per size class for 'Conference' pears for the 2015-2016 season for quality class A3 (Belgische Fruitveiling BFV, 2016, personal communication).

Table S7. Results of linear regression models assessing the effect of the distance to *Osmia* nesting boxes and year on pear fruit quality without outliers (n = 16). Model statistics degrees of freedom (df), F-values and p-values are given.

Table S8. Overview of species/families (or a higher taxonomic level*) that were identified in the limb beating samples in the pear trees and in the mixed hedgerow, and their considered role (pest, beneficial or indifferent).

Table S1. Products used and timing of interventions in the study pear orchard over the different years: (**A**) 2016 –(**B**) 2017 –(**C**) 2018.

(A)

Year	Purpose	Formulated Product (Active Ingredient)	Dose Rate (kg or l/ha LWA*) (l/ha for Herbicides)	Date
2016	Insect control	Sunspray 7E (paraffinic oil)	6.0	22/03/2016
		Decis 2.5 EC (deltamethrin)	0.4	13/03/2016
		Calypso 480 SC (thiacloprid)	0.25	13/03/2016
		Vertimec 18 EC (left side) (abamectin)	0.33	9/05/2016
		Vertimec 18 EC (right side) (abamectin)	0.33	26/05/2016
		Movento 100 SC (left side) (spirotetramat)	1.5	27/05/2016
	Fungal/bacterial diseases control	Mastana 500 SC (mancozeb)	2.66	13/04/2016
		Chorus 50 WG (cyprodinil)	0.3	28/04/2016
		Captan 80 WG (captan)	1.2	28/04/2016
		Captan 80 WG (captan)	1.2	2/05/2016
		Pomarsol 80 WG (thiram)	1.46	9/05/2016
		Captan 80 WG (captan)	1.2	26/05/2016
		Delan 70 WG (dithianon)	0.5	2/06/2016
		Geyser 250 EC (difenconazol)	0.1	2/06/2016
		Captan 80 WG (captan)	1.2	7-9/06/2016
		Delan 70 WG (dithianon)	0.5	14/06/2016
		Pomarsol 80 WG (thiram)	1.46	2/08/2016
		Bellis 38 WG (boscalid + pyroclostrobin)	0.5	10/08/2016
		Bellis 38 WG (boscalid + pyroclostrobin)	0.5	19/08/2016
		Geoxe 50 WG (fludioxonil)	0.33	5/09/2016
	Weed control	Lentipur 500 SC (chloortuloron)	1.0	20/06/2016
		Stomp Aqua 455 CS (pendimethalin)	1.0	20/06/2016
		Limurex 50 SC (linuron)	1.0	20/06/2016
	Growth regulation	Promalin 1.9 SL (gibberellic acid 4 and 7 (GA4+7) + 6-benzyladenine (6-BA))	0.33	24/04/2016
		Promalin 1.9 SL (gibberellic acid 4 and 7 (GA4+7) + 6-benzyladenine (6-BA))	0.2	28/04/2016
		Globaryll 100 SL (6-benzyladenine)	1.33	16/05/2016
		Fixor 100 SL (α-naphthyl acetic acid)	0.66	31/08/2016

* **Leaf Wall Area (LWA).** Since pear trees are a vertical crop, the product dose rate and water volume are expressed per ha LWA, as recommended by EPPO (guideline PP1/239(3) - Dose expression for plant protection products. 2020). The LWA is calculated by the number of trees x planting distance within the row (d) x treated tree height x 2 sides, and corresponded to 15,500 m2/ha soil surface in the trial orchard.

(B)

Year	Purpose	Formulated Product (Active Ingredient)	Dose Rate (kg or l/ha LWA*) (l/ha for Herbicides)	Date
2017	Insect control	Sunspray 7E (paraffinic oil)	6.0	10/03/2017
		Sunspray 7E (paraffinic oil)	6.0	24/03/2017
		Decis 2.5 EC (deltamethrin)	0.4	2/04/2017
		Calypso 480 SC (thiacloprid)	0.25	2/04/2017
		Isomate CLR ((E,E)-8,10-Dodecadien-1-ol, 1-Dodecanol, 1-Tetradecanol, (Z)-11-Tetradecen-1-yl acetate, (Z)-9-Tetradecen-1-yl acetate, (Z)-8-Tetradecen-1-yl acetate, (Z)-8-Tetradecen-1-ol)	800 dispensers/ha	1/05/2017
		Movento 100 SC (spirotetramat)	1.5	16/05/2017
		Movento 100 SC (spirotetramat)	1.5	20/05/2017
		Decis 2.5 EC (deltamethrin)	0.4	26/09/2017
		Calypso 480 SC (thiacloprid)	0.25	26/09/2017
	Fungal/bacterial diseases control	Syllit 400 SC (dodine)	0.8	24/03/2017
		Switch 62.5 WG (cyprodinil + fludioxonil)	0.5	4/04/2017
		Delan 70 WG (dithianon)	0.33	20/04/2017
		Delan 70 WG (dithianon)	0.33	16/05/2017
		Captan 80 WG (captan)	1.2	26/05/2017
		Geyser 250 EC (difenconazol)	0.1	6/06/2017
		Delan 70 WG (dithianon)	0.30	15/07/2017
		Captan 80 WG (captan)	1.2	29/07/2017
		Captan 80 WG (captan)	1.2	14/08/2017
		Bellis 38 WG (boscalid + pyroclostrobin)	0.5	28/08/2017
		Bellis 38 WG (boscalid + pyroclostrobin)	0.5	8/09/2017
		Switch 62.5 WG (cyprodinil + fludioxonil)	0.5	13/09/2017
	Weed control	Lentipur 500 SC (chloortuloron)	1.0	6/05/2017
		Stomp Aqua 455 CS (pendimethalin)	1.0	6/05/2017
		Limurex 50 SC (linuron)	1.0	6/05/2017

* **Leaf Wall Area (LWA).** Since pear trees are a vertical crop, the product dose rate and water volume are expressed per ha LWA, as recommended by EPPO (guideline PP1/239(3) - Dose expression for plant protection products. 2020). The LWA is calculated by the number of trees x planting distance within the row (d) x treated tree height x 2 sides, and corresponded to 15,500 m2/ha soil surface in the trial orchard.

(C)

Year	Purpose	Formulated Product (Active Ingredient)	Dose Rate (kg or l/ha LWA*) (l/ha for Herbicides)	Date
2018	Insect control	Sunspray 7E (paraffinic oil)	6.0	28/03/2018
		Decis 2.5 EC (deltamethrin)	0.4	10/04/2018
		Calypso 480 SC (thiacloprid)	0.25	10/04/2018
		Isomate CLR ((E,E)-8,10-Dodecadien-1-ol, 1-Dodecanol, 1-Tetradecanol, (Z)-11-Tetradecen-1-yl acetate, (Z)-9-Tetradecen-1-yl acetate, (Z)-8-Tetradecen-1-yl acetate, (Z)-8-Tetradecen-1-ol)	800 dispensers/ha	28/04/2018
		Movento 100 SC (spirotetramat)	1.5	25/05/2018
		Vertimec 18 EC (abamectin)	0.33	23/06/2018
	Fungal/bacterial diseases control	Syllit 400 SC (dodine)	0.8	4/04/2018
		Captan 80 WG (captan)	1.2	23/04/2018
		Captan 80 WG (captan)	1.2	11/05/2018
		Geyser 250 EC (difenconazol)	0.1	18/05/2018
		Chorus 50 WG (cyprodinil)	0.3	26/05/2018
		Pomarsol 80 WG (thiram)	1.66	5/08/2018
		Switch 62.5 WG (cyprodinil + fludioxonil)	0.5	20/08/2018
		Geoxe 50 WG (fludioxonil)	0.266	28/08/2018
	Weed control	Kyleo 240 SL (glyphosate)	2.5	4/04/2018
	Growth regulation	Globaryll 100 SL (6-benzyladenine)	1.33	11/05/2018
		Fixor 100 SL (α-naphthyl acetic acid)	0.66	18/08/2018
		Fixor 100 SL (α-naphthyl acetic acid)	0.66	26/08/2018

*** Leaf Wall Area (LWA).** Since pear trees are a vertical crop, the product dose rate and water volume are expressed per ha LWA, as recommended by EPPO (guideline PP1/239(3) - Dose expression for plant protection products. 2020). The LWA is calculated by the number of trees x planting distance within the row (d) x treated tree height x 2 sides, and corresponded to 15,500 m2/ha soil surface in the trial orchard.

Table S2. Composition of the mixed hedgerow at the border of the study pear orchard.

Woody Plant Species	Number of Woody Plants
Viburnum lantana (wayfarer)	17
Salix caprea (goat willow)	15
Viburnum opulus (guelder rose)	26
Ligustrum vulgare (common privet)	24
Corylus avellana (common hazel)	22
Frangula alnus (breaking buckthorn)	13
Cornus sanguinea (bloody dogwood)	7
*Fraxinus exelsior (*common ash*)*	2
Cornus mas (European cornel)	15
Sambucus nigra (European elder)	4
Alnus glutinosa (common alder)	7
Fagus sylvatica (common beech)	3
Acer campestre (field maple)	14
Cytisus scoparius (common broom)	5
Carpinus betulus (common hornbeam)	1

Table S3. Overview of insects observed visiting pear flowers during pear flowering in 2016 (**A**), and overview of the flower-visiting insects monitored during the transect walks after flowering in 2016-2018 (**B**).

(A)

Pollinator	Percent % (of Total Number of Flower-Visiting Insects Observed)
Apis melifera	39.8
Bombus spp.	2.7
Osmia cornuta	37.3
other solitary bees	20.2

(B)

Insect Observed	Number Observed
Andrena bimaculata	2
Andrena carantonica	2
Andrena cineraria	1
Andrena fulva	1
Andrena fuscipes	2
Andrena haemorrhoa	7
Andrena vaga	1
Anthophora plumipes	7
Apis mellifera	84
Bombus hypnorum	5
Bombus lapidarius	26
Bombus pascuorum	39
Bombus pratorum	20
Bombus terrestris	108
Chalcosyrphus eunotus	1
Chrysis spp.	1
Criorhina ranunculi	1
Doros profuges	1
Epistrophe melanostoma	2
Episyrphus balteatus	27

Eristalis arbustorum	52
Eristalis nemorum	1
Eristalis pertinax	12
Eristalis tenax	10
Eupeodes latifasciatus	1
Eupeodes luniger	3
Ferdinandea cuprea	1
Halictus rubicundus	2
Helophilus pendulus	1
Helophilus spp.	6
Lasioglossum malacharum	1
Lasioglossum spp.	1
Lucilia sericata	1
Maculinea spp.	1
Megachile centuncularis	2
Megasyrphus erraticus	2
Mesembrina meridiana	1
Myathropa florea	3
Nomada flava	3
Osmia cornuta	19
Pieris rapae	3
Scaeva pyrastri	1
Scaeva selenitica	1
Sphaerophoria batava	6
Sphaerophoria scripta	4
Syrphus ribesii	8
Tachina fera	3
Vanessa atalanta	1
Vanessa cardui	1
Vespa crabro	1
Vespula vulgaris	242
Volucella zonaria	1

Table S4. Overview of the plants visited for each functional group over the different years (after pear flowering): (**A**) 2016 –(**B**) 2017 –(**C**) 2018.

A:

Apis mellifera	Visit (%)	*Bombus* spp.	Visit (%)	Solitary Bees	Visit (%)	Syrphid Flies	Visit (%)
Chamaenerion angustifolium	3	*Chamaenerion angustifolium*	5	*Cornus sanguinea*	50	*Achillea millefolium*	20
Cornus sanguinea	46	*Cornus sanguinea*	11	*Phacelia tanacatifolia*	50	*Anthriscus sylvestris*	33
Geranium pyrenaicum	11	*Cotoneaster* spp.	1			*Bellis perennis*	2
Heracleum sphondylium	6	*Cytisus scoparius*	6			*Cirsium arvense*	15
Phacelia tanacatifolia	29	*Geranium pyrenaicum*	6			*Daucus carota*	2
Ranunculus repens	3	*Lamium album*	17			*Heracleum sphondylium*	7
Symphytum officinale	3	*Malva sylvestris*	5			*Lotus corniculatus*	2
		Papaver somniferum	2			*Scorzoneroides autumnalis*	4
		Phacelia tanacatifolia	18			*Solidago* spp.	13
		Prunus padus	1			*Sonchus* spp.	2
		Prunus serotina	2			*Symphytum officinale*	2
		Ranunculus repens	1				
		Symphytum officinale	4				
		Taraxacum agg.	1				
		Trifolium pratense	1				
		Trifolium repens	14				
		Viburnum opulus	1				
		Vicia cracca	2				

B:

Apis mellifera	Visit (%)	*Bombus* spp.	Visit (%)	Solitary Bees	Visit (%)	Syrphid Flies	Visit (%)
Cirsium arvense	17	*Bellis perennis*	1	*Bryonia dioica*	17	*Bellis perennis*	11
Phacelia tanacatifolia	17	*Centaurea jacea*	7	*Centaurea jacea*	17	*Centaurea jacea*	3
Rhamnus frangula	33	*Chamaenerion angustifolium*	7	*Chamaenerion angustifolium*	17	*Cirsium arvense*	16
Silene latifolia	17	*Cirsium arvense*	4	*Prunus padus*	17	*Daucus carota*	3
Taraxacum agg.	17	*Cytisus scoparius*	7	*Taraxacum* agg.	33	*Heracleum sphondylium*	13
		Geranium pyrenaicum	4			*Hypericum perforatum*	11
		Hypericum perforatum	1			*Phacelia tanacatifolia*	5
		Lamium album	6			*Taraxacum* agg.	39
		Linaria vulgaris	1				
		Lotus corniculatus	5				
		Malva sylvestris	16				
		Phacelia tanacatifolia	16				
		Prunus padus	1				
		Ranunculus repens	1				
		Rhamnus frangula	6				
		Silene latifolia	1				
		Taraxacum agg.	5				
		Trifolium pratense	5				
		Trifolium repens	5				
		Viburnum opulus	1				

C:

Apis mellifera	Visit (%)	*Bombus* spp.	Visit (%)	Solitary Bees	Visit (%)	Syrphid Flies	Visit (%)
Cornus sanguinea	17	*Convolvulus sepium*	12	*Cornus sanguinea*	33	*Bellis perennis*	2
Galium aparine	17	*Cornus sanguinea*	12	*Rubus* spp.	33	*Convolvulus arvensis*	2
Heracleum sphondylium	50	*Cytisus scoparius*	29	*Taraxacum* agg.	33	*Convolvulus sepium*	5
Hypochoeris radicata	17	*Epilobium hirsutum*	29			*Cornus sanguinea*	10
		Hypericum perforatum	6			*Heracleum sphondylium*	29
		Hypochoeris radicata	6			*Hypericum perforatum*	2
		Taraxacum agg.	6			*Lolium perenne*	2
						Pyrus communis	24
						Rubus spp.	5
						Taraxacum agg.	7
						Urtica dioica	10

Table S5. Results of linear regression models assessing the effect of pollination treatment (bagged vs open flowers) and year on pear fruit quality without outliers (n = 17). Model statistics degrees of freedom (df), F-values and p-values are given.

Factor	df	F	p
Pollination treatment	1	100.2	< 0.001
Year	2	187.7	< 0.001
Pollination treatment:Year	2	8.1	< 0.001

Table S6. Medium price per size class for 'Conference' pears for the 2015-2016 season for quality class A3 (Belgische Fruitveiling BFV, 2016, personal communication).

Size Class	Price (€/kg)
45-50 mm	0.220
50-65 mm	0.329
55-65 mm	0.423
60-65 mm	0.470
65-70 mm	0.551
70-75 mm	0.611
75-80 mm	0.623

Table S7. Results of linear regression models assessing the effect of the distance to *Osmia* nesting boxes and year on pear fruit quality without outliers (n = 16). Model statistics degrees of freedom (df), *F*-values and *p*-values are given.

Factor	df	*F*	*p*
Distance	1	30.9	< 0.001
Year	2	98.7	< 0.001
Distance:Year	2	2.6	0.018

Table S8. Overview of species/families (or a higher taxonomic level*) that were identified in the limb beating samples in the pear trees and in the mixed hedgerow, and their considered role (pest, beneficial or indifferent).

Species/Family or Higher Taxonomic Level	Order	Role
Acarina	Acarina	indifferent
Anaspis sp.	Coleoptera	indifferent
Anthocoris nemoralis	Hemiptera	beneficial
Anthocoris nemorum	Hemiptera	beneficial
Aphidoidea	Hemiptera	pest
Aphis fabae	Hemiptera	pest
Aphis frangulae	Hemiptera	indifferent
Aphis lantanae	Hemiptera	indifferent
Araneomorphae	Araneae	beneficial
Araneus diadematus	Araneae	beneficial
Araneus sp.	Araneae	beneficial
Aulacorthum solani	Hemiptera	indifferent
Barypeithes araneiformis	Coleoptera	pest
Bdellidae	Acarina	beneficial
Bembibion sp.	Coleoptera	beneficial
Blepharidopterus angulatus	Hemiptera	beneficial
Bruchidius sp.	Coleoptera	pest
Cacopsylla pyri	Hemiptera	pest
Calvia quatuordecimguttata	Coleoptera	beneficial
Campylomma verbascii	Hemiptera	pest
Campyloneura virgula	Hemiptera	beneficial
Cantharis sp.	Coleoptera	beneficial
Cardiastethus fasciiventris	Hemiptera	beneficial
Cecidomyiidae	Diptera	pest
Chalcidoidea	Hymenoptera	beneficial
Chalcidoidea (hyperparasitoid)	Hymenoptera	pest
Chironomus plumosus	Diptera	indifferent
Chloropidae sp.	Diptera	indifferent
Chrysoperia carnea	Neuroptera	beneficial
Cicadellidae	Hemiptera	pest
Coccinellidae	Coleoptera	beneficial
Coleoptera	Coleoptera	indifferent
Collembola	Collembola	indifferent
Coreidae	Hemiptera	pest
Crambidae	Lepidoptera	indifferent
Crossocerus exiguus	Hymenoptera	indifferent
Curculionidae	Coleoptera	pest
Delphacidae	Hemiptera	pest
Demetrias atricapillus	Coleoptera	beneficial
Deraeocoris lutescens	Hemiptera	beneficial
Deraeocoris ruber	Hemiptera	beneficial
Dicyphus sp.	Hemiptera	beneficial
Diptera	Diptera	indifferent
*Drosophila suzukii***	Diptera	indifferent

Drosophilidae	Diptera	indifferent
Elateridae	Coleoptera	pest
Enoplognatha sp.	Araneae	beneficial
Euceraphis betulae	Hemiptera	pest
Eupoedes sp.	Diptera	beneficial
Forficulidae	Dermaptera	beneficial
Formica rufibarbis	Hymenoptera	pest
Formicidae	Hymenoptera	pest
Gastropoda	Gastropoda	pest
Gelis sp.	Hymenoptera	pest
Geometridae	Lepidoptera	pest
Gonocerus acuteangulatus	Hemiptera	pest
Gracillaridae	Lepidoptera	pest
Halictidae	Hymenoptera	beneficial
Harmonia axyridis	Coleoptera	beneficial
Hemerobiidae	Neuroptera	beneficial
Heteroptera	Hemiptera	indifferent
Heterotoma planicornis	Hemiptera	beneficial
Ichneumonoidae (parasitoid)	Hymenoptera	beneficial
Isopoda	Isopoda	indifferent
Laelapidae	Acarina	beneficial
Lagria hirta	Coleoptera	indifferent
Lasius niger	Hymenoptera	pest
Latridiidae sp.	Coleoptera	indifferent
Lepidoptera	Lepidoptera	pest
Liocoris tripustulatus	Hemiptera	indifferent
Lygaeidae	Hemiptera	indifferent
Lygocoris pabulinus	Hemiptera	pest
Malacocoris chlorizans	Hemiptera	beneficial
Microlepidoptera	Lepidoptera	pest
Micromus variegatus	Neuroptera	beneficial
Miridae	Hemiptera	pest
Nabidae sp.	Hemiptera	beneficial
Noctuidae	Lepidoptera	indifferent
Notostira elongata	Hemiptera	indifferent
Opiliones sp.	Araneae	beneficial
Oribatida sp.	Acarina	indifferent
Orius sp.	Hemiptera	beneficial
Orthops kalmii	Hemiptera	pest
Otiorhynchus sp.	Coleoptera	pest
Otiorhynchus tenebricosus	Coleoptera	pest
Oulema melanopus	Coleoptera	indifferent
Oxybelus uniglumis	Hymenoptera	indifferent
Palomena prasina	Hemiptera	pest
Panorpidae	Mecoptera	beneficial
Pentatomidea	Hemiptera	pest
Phyllobius sp.	Coleoptera	pest
Piezodorus lituratus	Hemiptera	indifferent
Pilophorus perplexus	Hemiptera	beneficial
Protapion fulvipes	Coleoptera	pest
Protapion sp.	Coleoptera	indifferent
Psocoptera	Psocoptera	indifferent
Psyllidae	Hemiptera	pest
Psylliodes sp.	Coleoptera	pest
Psyllobora vigintiduopunctata	Coleoptera	beneficial
Psyllopsis fraxini	Hemiptera	indifferent
Pterophoridae	Lepidoptera	indifferent

Selandria serva	Hymenoptera	indifferent
Sitona sp.	Coleoptera	pest
Sphaerophoria scripta	Diptera	beneficial
Staphylinidae	Coleoptera	beneficial
Stenodema laevigata	Hemiptera	indifferent
Stephanitis takeyai	Hemiptera	indifferent
Tachyporus sp.	Coleoptera	beneficial
Tenthredinidae	Hymenoptera	pest
Theridion sp.	Araneae	beneficial
Theridion varians	Araneae	beneficial
Thomisidae	Araneae	beneficial
Thysanoptera (Thrips)	Thysanoptera	pest
Tipulidae	Diptera	pest
Trigonotylus caelestialium	Hemiptera	indifferent
Trombidiidae	Acarina	beneficial

* While well-known pests or beneficial arthropods were identified to species or family level based on morphological characteristics, other specimens (mostly considered as indifferent) were only classified into the family or an even higher taxonomic level for this study. ** Although *D. suzukii* is a serious pest in soft fruits, it is not considered as a pest for pome fruit.
